# Aligned electrospun fibers for neural patterning

**DOI:** 10.1007/s10529-017-2494-z

**Published:** 2018-01-08

**Authors:** Erfan Soliman, Fabio Bianchi, James N. Sleigh, Julian H. George, M. Zameel Cader, Zhanfeng Cui, Hua Ye

**Affiliations:** 1 Institute of Biomedical Engineering, Old Road Campus Research Building, University of Oxford, Oxford, OX3 7DQ UK; 2 The Weatherall Institute of Molecular Medicine, University of Oxford, John Radcliffe Hospital/Headley Way, Oxford, OX3 9DS UK; 3 Present Address: Sobell Department of Motor Neuroscience and Movement Disorders, Institute of Neurology, University College London, London, WC1N 3BG UK

**Keywords:** Electrospinning, Fibre network, Glioma cells, Neural network, Neuroblastoma, Polycaprolactone

## Abstract

**Objectives:**

To test a 3D approach for neural network formation, alignment, and patterning that is reproducible and sufficiently stable to allow for easy manipulation.

**Results:**

A novel cell culture system was designed by engineering a method for the directional growth of neurons. This uses NG108-15 neuroblastoma x glioma hybrid cells cultured on suspended and aligned electrospun fibers. These fiber networks improved cellular directionality, with alignment angle standard deviations significantly lower on fibers than on regular culture surfaces. Morphological studies found nuclear aspect ratios and cell projection lengths to be unchanged, indicating that cells maintained neural morphology while growing on fibers and forming a 3D network. Furthermore, fibronectin-coated fibers enhanced neurite extensions for all investigated time points. Differentiated neurons exhibited significant increases in average neurite lengths 96 h post plating, and formed neurite extensions parallel to suspended fibers, as visualized through scanning electron microscopy.

**Conclusions:**

The developed model has the potential to serve as the basis for advanced 3D studies, providing an original approach to neural network patterning and setting the groundwork for further investigations into functionality.

## Introduction

Tissue-engineered platforms provide specialized mechanical and environmental cues in vitro for dissociated cells to grow, expand, and differentiate in conditions resembling in vivo settings. Such tissue environments can be invaluable for understanding cellular processes, functional development, and for more disease-relevant drug testing.

Axons and dendrites of neurons in both the central and peripheral nervous systems (CNS and PNS) are long and thin cellular structures that often show distinct patterns of alignment (Catani et al. 2002). Patterning of substrates on 2D surfaces can be used to provide guidance to growing neurons. Common techniques include micro-contact printing with chemo attractant protein inks (James et al. 2000), micro-pillar construction for substrate patterning (Dowell-Mesfin et al. 2004), aligned deposited fibers (Wu et al. 2015) and coating with rough surfaces (Kim et al. 2006). Neurons preferentially respond to environments patterned to better resemble in vivo conditions, even without biological cues (Richardson et al. 2011).

3D patterning of gels and constructs has also been used to develop engineered neural tissue, reproducing the alignment found in the CNS and PNS. Methods used include aligned co-culture models of neurons and oligodendrocytes or Schwann cells (Daud et al. 2012), gel-fiber scaffolds (Tang-Schomer et al. 2014) and tethered gels aligned by contractile forces (Georgiou et al. 2013). However, methods for 3D patterning of neural networks are often not easily reproducible nor sufficiently stable to allow for easy manipulation (Georgiou et al. 2013).

More generally, patterning of neural networks can provide a means of enhancing their responsiveness, through increases in average firing rates of active neurons as well as in the proportion that are active out of the total population to begin with (Chang et al. 2001). Previous studies have confirmed the important role that patterning can play in augmenting neural outgrowth, whether through directional guidance (Yang et al. 2005) or through the loading and transfer of factors and proteins (Chang et al. 2003). Furthermore, in vitro scaffolds capable of mimicking the chemical, mechanical, and topographical characteristics of the extracellular matrix (ECM) are beneficial for assisting cell interactions (Koh et al. 2008). Electrospinning is an example of one method capable of producing polymer scaffolds with structurally comparable architecture to the ECM of tissue (Koh et al. 2008).

Here, we present an in vitro culture system of suspended, aligned, electrospun gelatin and polycaprolactone (PCL) fibers. Previously, suspended electrospun fibers have been used in wound healing models (Sheets et al. 2013), but here we apply them to patterning of neural networks formed by cultured neurons. Fibers are electrospun onto custom-geometry inserts, designed to allow for the cells growing on them to be completely suspended in culture medium. This system removes interactions between the cells and the underlying surface, thereby providing a 3D environment.

## Materials and methods

### Cell culture and differentiation

The rodent neuroblastoma × glioma hybrid cell line NG108-15 (Sigma-Aldrich) was cultured in Dulbecco’s Modified Eagle Medium (DMEM) containing 10% (v/v) fetal bovine serum (FBS) and 1% (w/v) penicillin/streptomycin (P/S) in Corning T75 or T25 flasks. Cells were incubated at 37 °C and 5% CO_2_ in air and passaged every 4–6 days (when ~ 70–90% confluent). Passaging was carried out by mechanical detachment, as these cells adhere lightly to the culture surface and do not require trypsin: old medium was aspirated, 5 ml PBS was added, and the flask was gently tapped against the tissue culture hood until cells were no longer adhered. The mixture was then transferred to a 15 ml tube and centrifuged for 5 min at 1000×*g*. The PBS was aspirated and the cell pellet re-suspended in 1 ml new culture medium before seeding. Differentiation was induced through serum starvation and the addition of dimethyl sulfoxide (DMSO) once cells were ~ 60% confluent. The differentiation medium was made up of 1.5% (v/v) DMSO, 1% (v/v) P/S and 0.5% (v/v) FBS in DMEM (Schubert et al. 1971).

### Electrospinning, growth chambers, and fiber coating

**Fig. 1:**
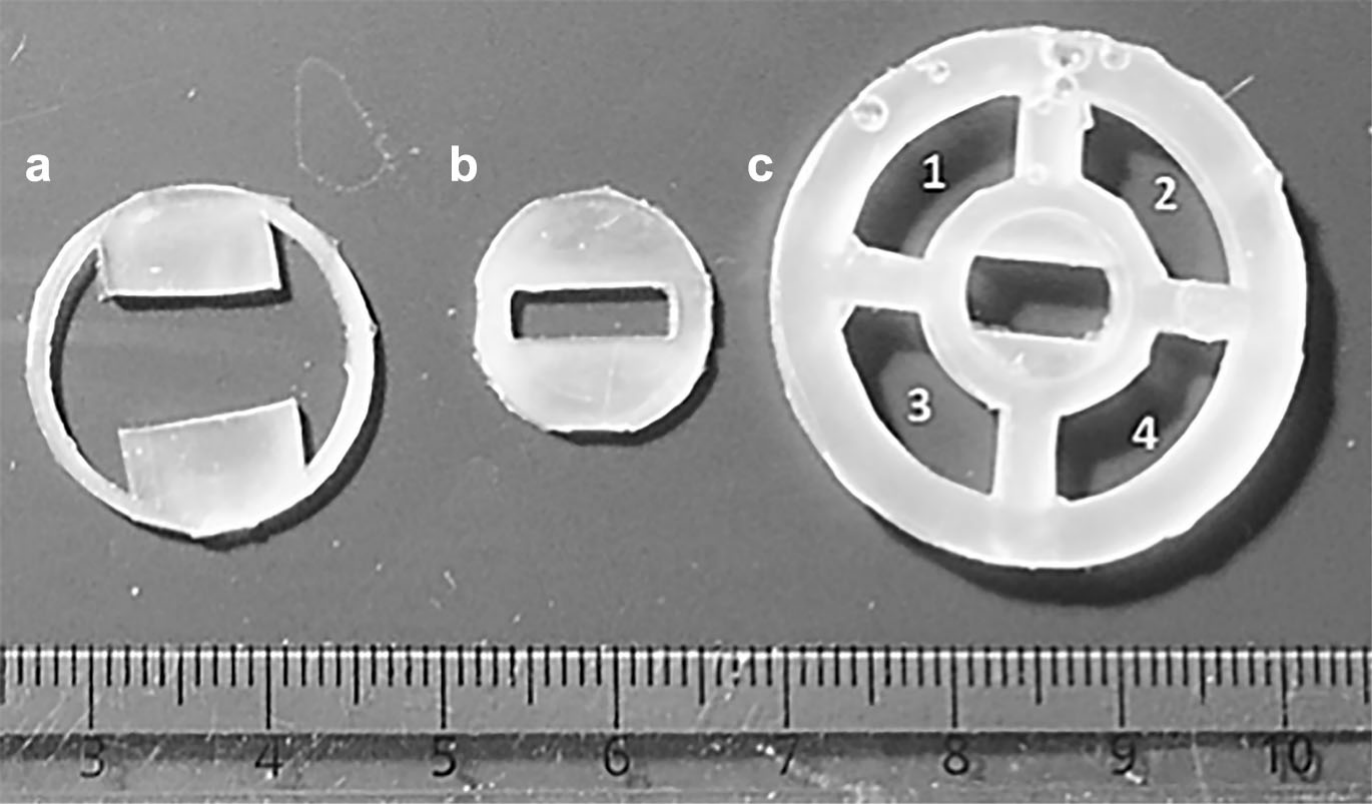
PDMS inserts used for assembling cell culture chambers, lettered in order of placement within the imaging dish (with A at the bottom, B in the middle, and C on top)

To prepare fiber growth chambers (Soliman et al. 2017), three silicone pieces were made with polydimethylsiloxane in such a way as to allow them to fit, stacked on top of one another, inside an Ibidi *µ*-dish (Ibidi) (Fig. 1). The polymer solution used for electrospinning was made by dissolving gelatin/polycaprolactone (PCL) (70:30, w/w) (Sigma-Aldrich) in hexafluoro-2-propanol (Apollo Scientific), to make 8% (w/v). Fibers were electrospun across a rectangular gap cut into the middle silicone piece, and a rotating drum collector was used to ensure fiber alignment during collection. The three silicone pieces were stacked to form the growth chamber, with fibers tightly held in place by the top piece, which presses down onto the assembly. This allows fibers to be completely suspended in culture medium while maintaining stability during manipulation and imaging.

Fibers were coated with fibronectin to enhance cell attachment. To do so, samples were incubated for 2 h at room temperature with 2 µg fibronectin/ml solution extracted from bovine plasma before washing with phosphate-buffered saline (PBS). Samples were sterilized under UV before cell culture.

### Immunostaining

After fixing in 3.7% (v/v) paraformaldehyde, cells were permeabilised and blocked in PBS containing 0.1% (v/v) Tween 20 0.2% (v/v) Triton-X, and 5% (w/v) bovine serum albumen (BSA) for 1 h at 37 ºC and washed three times in PBS (Jamur and Oliver 2010). *β*-III Tubulin, a neuron-specific microtubule protein, was stained using rabbit anti-Tuj1 (Abcam) in combination with a goat anti-rabbit secondary antibody conjugated with AlexaFluor 488 (green) fluorescent dye (Life Technologies).

### Scanning electron microscopy (SEM)

For SEM, samples were dehydrated by sequential immersion in ascending concentrations of ethanol (30, 50, 75, 90 and 100% (v/v) in water) for 5 min per concentration, followed by critical point drying in hexamethyldisilazane for 10 min. Samples were subsequently transferred onto carbon tape and attached to SEM sample holders. A Quorum SC7620 Mini Sputter Coater Glow Discharge System was used for gold–palladium coating (for 15 s) of fiber samples, in order to enable the use of the equipment in high vacuum mode. Fiber characterization and cell attachment images were obtained using a Carl Zeiss Evo LS15 VP-scanning electron microscope.

### Imaging and analysis

All bright-field and fluorescent microscopy was carried out using a Nikon inverted fluorescent microscope, and microscope settings were kept unchanged across related samples. Quantitative image analysis was carried out using batch-measuring tools of imaging software ImageJ/FIJI (SciJava), and results were calculated by averaging data from triplicate experiments.

### Cell alignment measurements

Nuclei aspect ratios were measured by taking the ratio of the longest to the shortest dimension of the circular nuclear structure. Neurite angles were measured by taking the angle made between neurites and the horizontal. The standard deviations of these angles were used as a measure of cell alignment, a lower value indicating greater directionality.

### Numerical and statistical analysis

GraphPad Prism 6 software was used for data and statistical analyses. All data sets were tested for normality using the D’Agostino & Pearson omnibus normality test (the Shapiro–Wilk or Kolmogorov–Smirnov normality tests were used to supplement). For statistical analysis, tests were chosen based on normality of the data set and the number of separate versus matched samples, with P values < 0.05 considered statistically significant (*P < 0.05, **P < 0.01, ***P < 0.001, ****P < 0.0001). Data was collected from three areas of each sample, from which at least three sub-areas were taken for measurements. All graphs display mean ± SD.

## Results

### Cell response to fibers and alignment

**Fig. 2:**
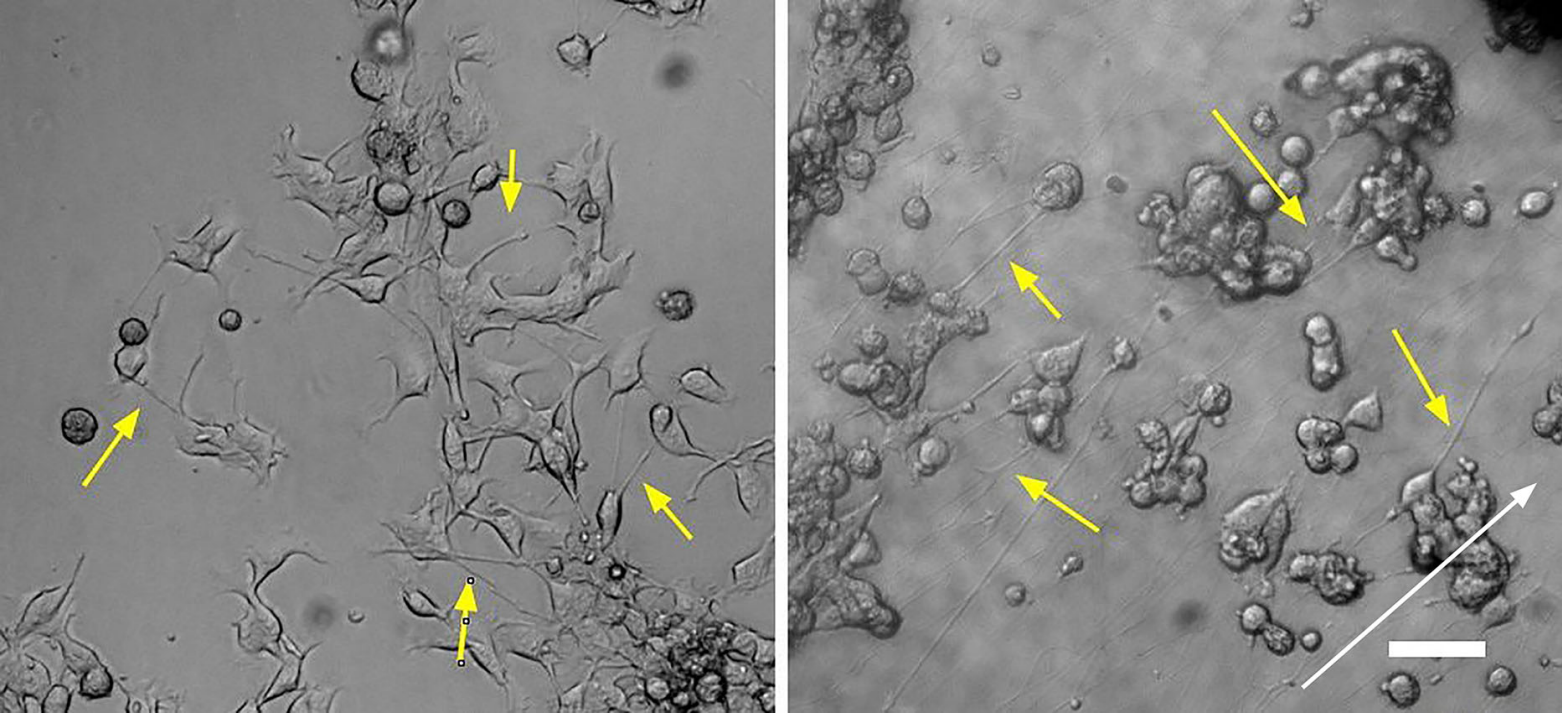
NG108-15 cells cultured for 96 h in proliferation medium on an uncoated flask (left) and on fibers (right). Yellow arrows highlight neurite extensions and white arrow indicates fiber direction. *Scale bar* 100 µm

**Fig. 3:**
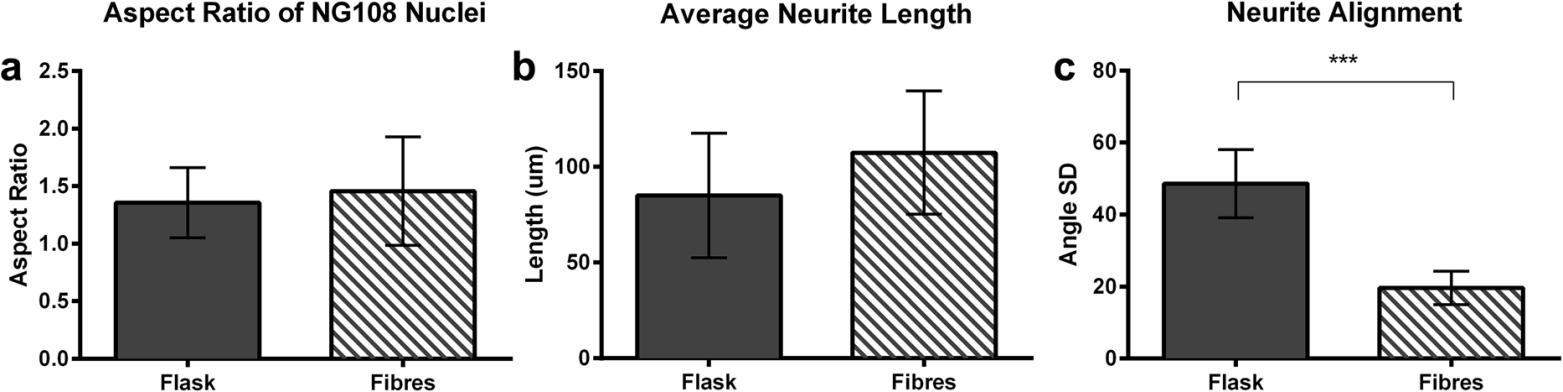
**a** Aspect ratio of NG108-15 nuclei (a value of 1 indicates a round object and values > 1 indicate an elongated object). **b** NG108-15 neurite lengths on untreated culture flasks and aligned fibers. **c** NG108-15 neurite extension angle standard deviations, cultured for 96 h on proliferation medium. Three samples per condition and > 15 measurements per sample were analyzed using an unpaired *t* test

Cells were fully viable in both growth and differentiation media for up to 5 days. Figure 2 displays NG108-15 cells cultured on fibers and on an uncoated flask surface, with yellow arrows indicating neurite extensions, and white arrow indicating fiber direction. When adhered to fibers, NG108-15 cells do not undergo significant morphological transformations, and do not spread and stretch along the fibers. The cells retain a roughly round central soma, extending neurites along fibers with little deformation of the cell body. Figure 3a summarizes measurements of nuclear aspect ratios, showing no significant difference between samples grown on fibers compared to those on flasks. This indicates that cells retain their neural morphology. Figure 3b shows a similar finding for neurite lengths, with no significant differences between the flask and fiber conditions.

For NG108-15 alignment analysis, the standard deviations of neurite projection angles—the angle between projections and the cell body—were calculated. Based on Fig. 3c, it is evident that fibers helped to guide neurite extensions, as angle standard deviations are significantly lower on fibers than on control flasks. Additionally, fiber orientations exhibit comparably low standard deviations (Soliman et al. 2017), suggesting that neurons extended neurites along fibers, without producing tensile forces strong enough to pull them out of alignment.

### Fibronectin fiber coating for NG108-15 attachment

**Fig. 4:**
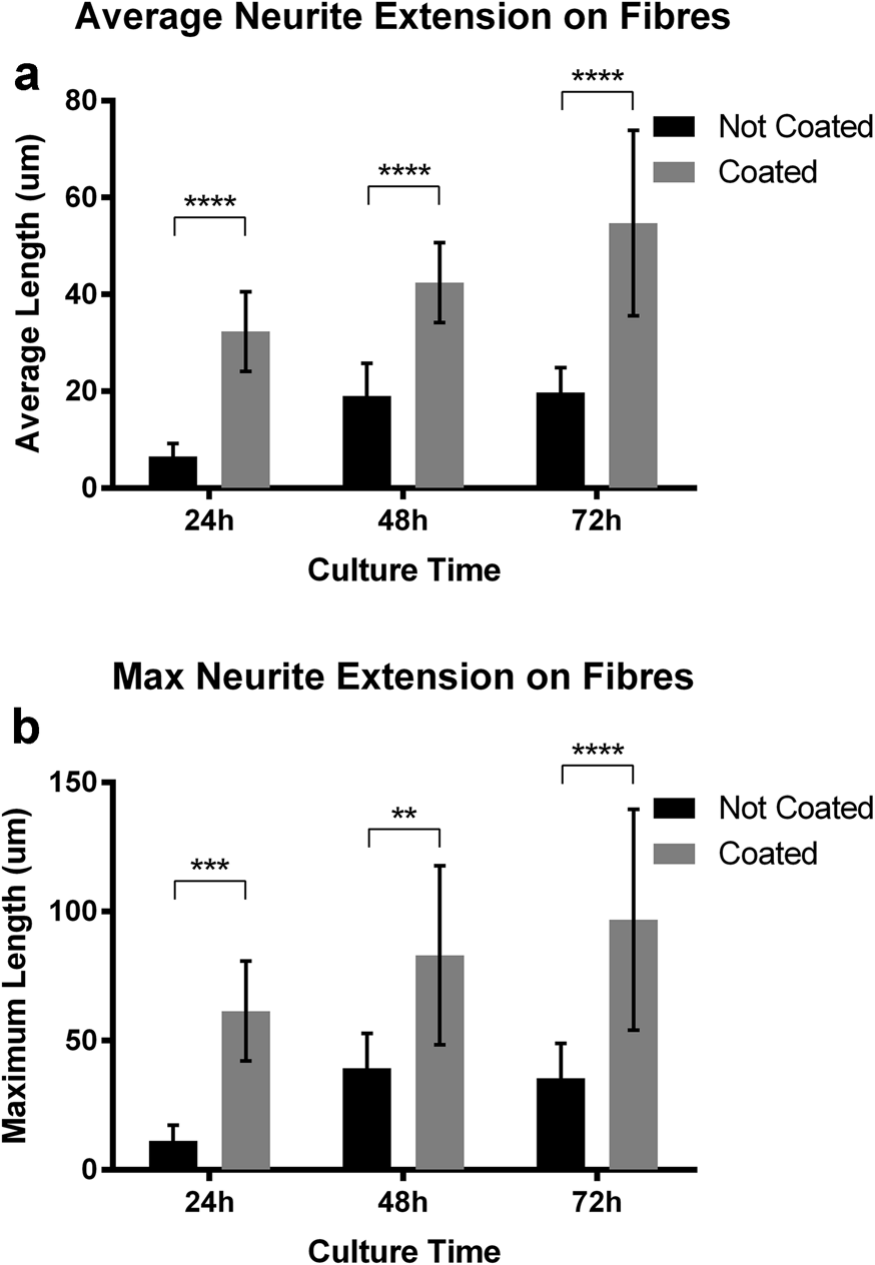
Average (**a**) and maximum (**b**) neurite extension on normal and fibronectin-coated fibers in growth medium. Cells were analyzed at 24, 48, and 72 h post plating. Three samples and > 20 measurements from three 250 × 250 pixel squares per sample were analyzed using a 2way ANOVA + Sidak’s multiple comparisons test

The effect of using a fibronectin coating was also examined by quantifying the neurite extension of NG108-15 cells grown on both coated and uncoated fibers in normal culture medium. Figures 4a and b illustrate significantly greater neurite extension (both average and maximum) on fibronectin-coated fibers. Fibronectin serves a key role in neurite growth through its interaction with *α*5*β*1 integrin receptors, reinforced by evidence of significantly decreased neurite extension on fibronectin following the blocking of these receptors (Tonge et al. 2012; Mukhatyar et al. 2011). These findings are in line with our results. Neurite extensions of NG108-15 cultures are shown in Fig. 2, with outgrowth apparent for cells cultured on coated fibers. This platform therefore provides a potential basis for directional neurite extension experiments that could examine the innervation patterns of neural networks.

### Cell spread and differentiation on fibers

**Fig. 5:**
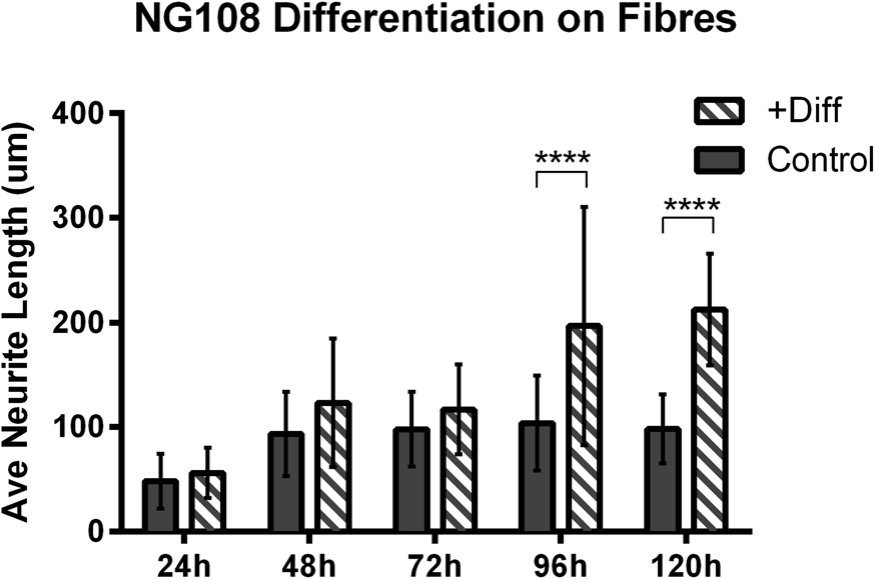
Average NG108-15 neurite length on fibers for cells in culture medium (control) and cells exposed to differentiation medium. Two samples per category and > 50 measurements per sample were analyzed using a 2way ANOVA + Sidak’s multiple comparisons test

Figure 5 summarizes the differences in average neurite length between cells grown in regular culture medium and cells exposed to differentiation medium (both grown on fibronectin-coated fibers). This indicates that average neurite extension increases following exposure to differentiation medium. Furthermore, qualitative observations (data not shown) reveal that cell density decreases following exposure to differentiation medium, preventing the typically observed clustering, and eventual death, of neurons growing on fibers for longer than 96 h. During differentiation, cells exit proliferative modes, and thus cell numbers remain constant. The proposed engineered platform is thus capable of supporting neuronal differentiation and network formation using medium-suspended fibers that allow for growth and 3D interaction.

**Fig. 6:**
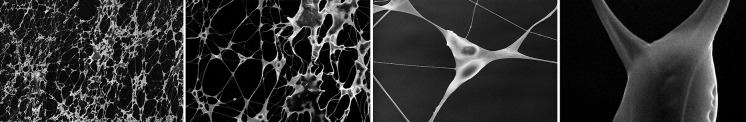
SEM images of NG108-15 cells on fibers, showing neurite network formation. Cells were cultured for 72 h in differentiation medium. Cell clustering is clearly visible in the second and third pictures. Magnification left to right: 560×, 1500×, 3000×, 17,000×

**Fig. 7:**
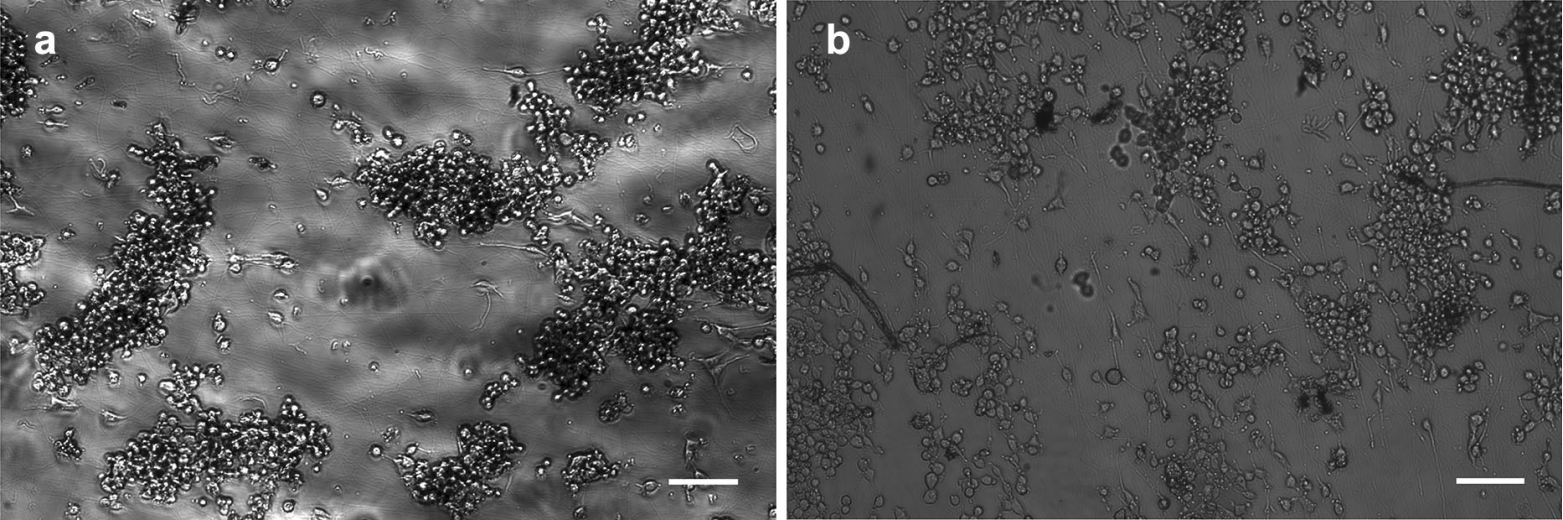
NG108-15 neurons growing on fibers after 120 h on (**a**) regular culture medium and (**b**) differentiation medium. *Scale bar* 200 µm

SEM imagery of cells differentiated on fibers for 2–3 days can be seen in Fig. 6. Specifically, the characteristics of roughly round central somas and neurite extensions along fibers are notable. Additionally, a complex neurite network may be observed in the first two images, indicative of interactions between cells that are able to differentiate on fibers (as opposed to those simply proliferating as non-differentiated cells). Figure 7 depicts neurons growing on fibers in culture medium and differentiation medium, showing that cells remain viable for up to 120 h.

## Discussion

A novel culture method was developed, with the ultimate intent of using suspended electrospun fibers for neural network patterning in vitro, to guide the direction of cells while maintaining natural morphology and cell characteristics. The use of suspended fibers allowed for observation of cell–cell and cell–fiber interactions within a 3D environment. In order to reliably develop and test this model, a three-piece culture chamber, assembled inside an imaging plate, was used to keep fibers from detaching during cell growth, transport, and manipulation.

The response to fibers was evaluated by looking at cell alignment and morphology, noting greater than 50% alignment for neurites on fibers compared to flasks. Further, there was no indication of significant cell deformation, as demonstrated through quantification of nuclear aspect ratios.

NG108-15 differentiation on fibers was also studied, with differentiation resulting in continued neurite extension along fibers, with a 50–100% increase in average neurite length, and a decrease in cell density. This result demonstrates that the culture system can be successfully used to influence directionality of neural networks, a novel approach in this area of tissue engineering. Additionally, cells exhibited enhanced neuronal growth on fibronectin-coated fibers, with significant increases in both average and maximum neurite extension.

Overall, favorable characteristics for cell culture were identified. Cell growth on electrospun fibers was achieved, with the alignment of fibers successfully retained by cells throughout development and differentiation. Further investigation into how suspended fibers may be used for more precise manipulation of culture outcomes will provide a strong basis for the development of functional neural networks in vitro.
